# *Apidologie* 50 years

**DOI:** 10.1007/s13592-020-00797-8

**Published:** 2020-08-19

**Authors:** Christiane Courant, Gudrun Koeniger, Klaus Hartfelder

**Affiliations:** 1 Eco-hameau d’Andral, F-46300 Le Vigan, France; 2 Biozentrum, Universität Würzburg, Am Hubland, D-97074 Würzburg, Germany; 3 Faculdade de Medicina de Ribeirão Preto, Universidade de São Paulo, Av. Bandeirantes 3900, Ribeirão Preto, SP 14049-900 Brazil

**Keywords:** Jean Louveaux, Friedrich Ruttner, INRA, Deutscher Imkerbund, Bee research

## Abstract

Since its foundation, *Apidologie* has steadily gained recognition as a journal that reports results from high-quality scientific research on the biology of bees, and this means Apidae in general, not only on its most prominent species, the Western honey bee, *Apis mellifera*. All started 50 years ago in a conversation between two eminent scientists, Jean Louveaux, director of one of INRA’s bee research unit in Bures-sur-Yvette and editor of the French *Annales de l’Abeille*, and Friedrich Ruttner, director of the Bee Research Institute in Oberursel and editor of the German *Zeitschrift für Bienenforschung*, where they discussed the possibility of merging these two journals to create an international bee research journal. Here, we take *Apidologie*’s 50th anniversary as an opportunity to provide our readers with background information on the journal’s history, especially on the persons and their contributions along this journey.

## Introduction

Nowadays, in the era of online and open-access publishing, scientific journals are frequently created by publishers who then search for and assign editors. Fifty years ago, when *Apidologie* had its birth date, the situation of scientific journals in biology was entirely different. For many decades, beekeepers, birdwatchers (ornithologists), and many educated citizens were interested in the newest scientific results in their special field of interest, and many of them read and paid for journals covering a broad array of subjects within these areas.

Among these were the French *Annales de l’ Abeille* and the German *Zeitschrift für Bienenforschung*, which served for many years the above-mentioned demand and had a wide distribution, both among bee scientists and beekeepers. However, with the fast development and progress of scientific techniques, it became increasingly difficult for beekeepers to understand scientific publications. Accordingly, many of those subscribers gave up reading the more science-oriented articles, and finally stopped ordering the journals. Hence, with decreasing subscribers, the economic basis became more and more risky for these two leading honey bee journals from France and Germany, and the proposal to merge was a reasonable move.

Here we review the history of *Apidologie*, from its beginnings until present times, presenting the persons and institutions and their contributions to the journal’s history. By providing glimpses into backstage events, we take this milestone 50-year anniversary as a unique opportunity to bring the journal closer to its current readers in the bee research community.

## The *Apidologie* idea and its realization

**Figure 1.**
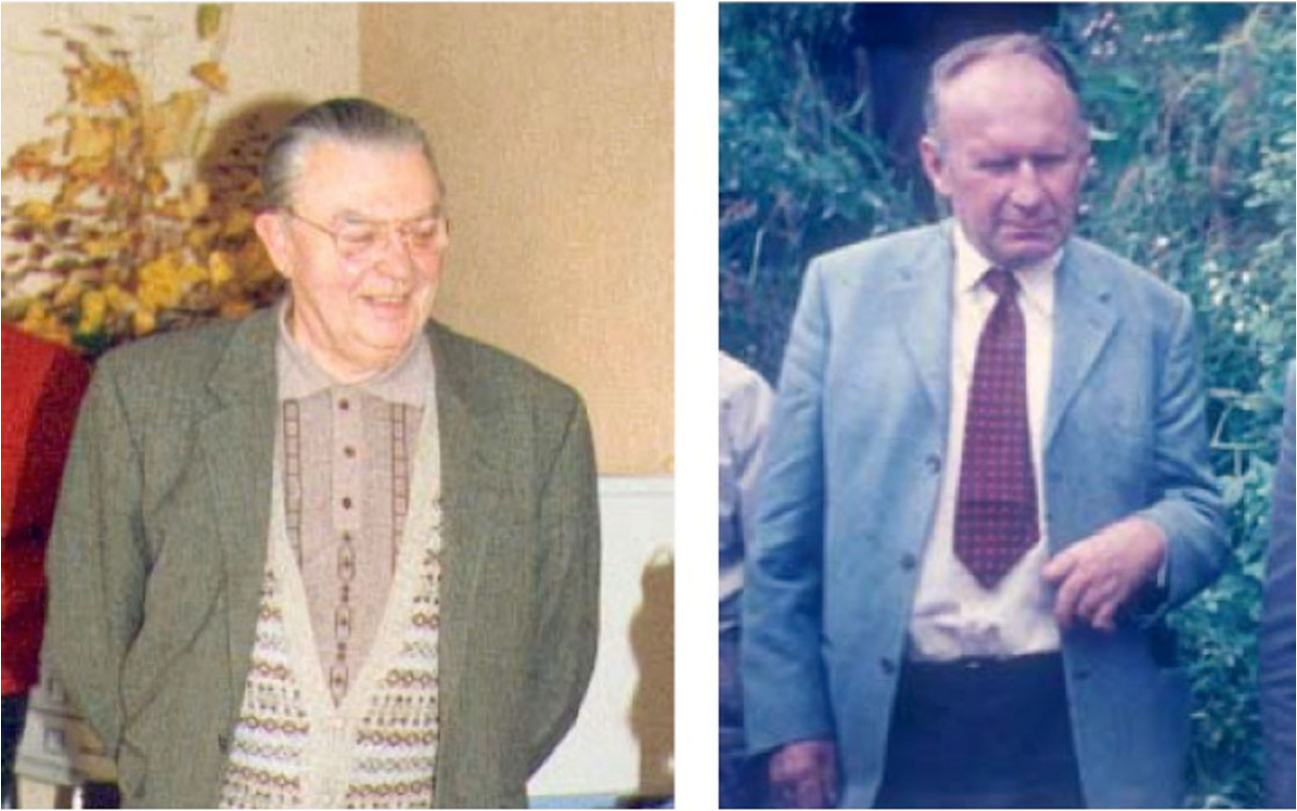
Jean Louveaux (left) and Friedrich Ruttner (right), the two founders of *Apidologie*.

Already for a long time, the two idealizers of *Apidolgie*, Jean Louveaux and Friedrich Ruttner (Figure 1), were close friends, and they often met at scientific and beekeeping honeybee congresses. Jean Louveaux was French and Director of the *Station de Recherches sur l’Abeille et les Insectes Sociaux* in Bures-sur-Yvette in France, and research unit of the *Institut national de la recherche agronomique* (INRA). He also was the editor of *Annales de l’Abeille*, which had its first issue published in 1958 by INRA. Friedrich Ruttner was Austrian and Director of the *Institut für Bienenkunde* in Oberursel near Frankfurt, Germany, and he was also the editor of the *Zeitschrift für Bienenforschung* that had its origins in 1950. The journal was published by the *Arbeitsgemeinschaft für Bienenkunde e.V*. in association with the *Deutsche Imkerbund* (DIB).

**Figure 2.**
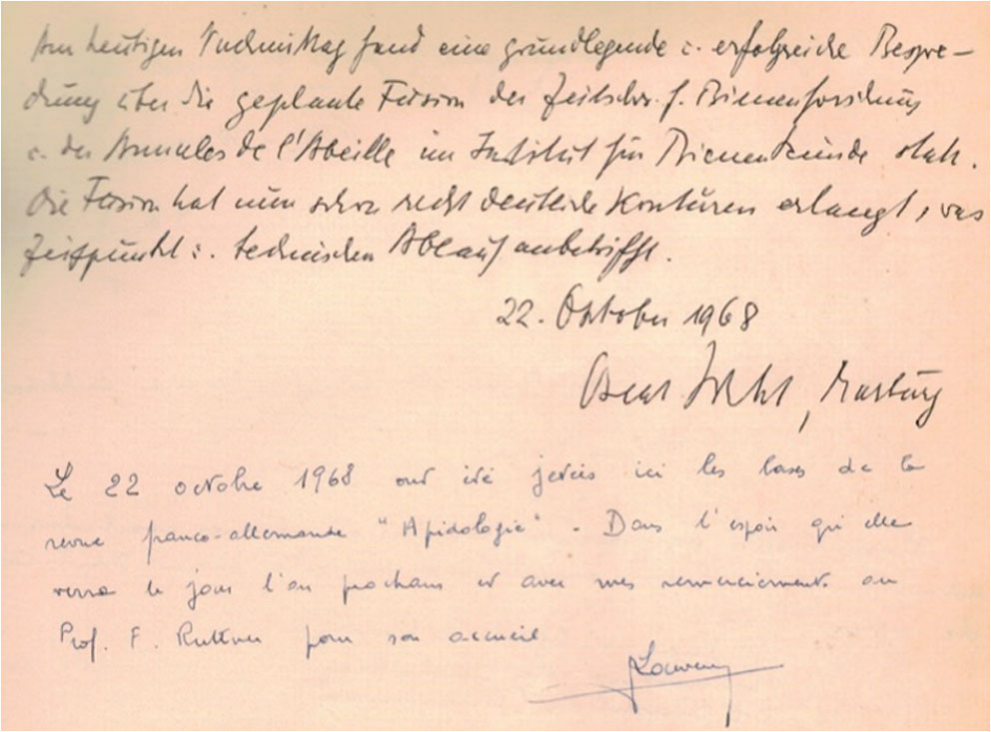
Note from the guestbook of the bee research institute in Oberursel, Germany, recording the meeting on October 22, 1968, where *Apidologie* was officially created. The note is signed by Oskar Wahl and Jean Louveaux. The German text reads: “Am heutigen Nachmittag fand eine grundlegende u. erfolgreiche Besprechung über die geplante Form der Zeitschr. f. Bienenforschung u. der Annales de l’Abeille im Institut für Bienenkunde statt. Die Form hat nun schon recht deutliche Konturen erlangt, was Zeitpunkt u. technischen Ablauf anbelangt.” The French text says: “Le 22 octobre 1968 ont été jetées ici les bases de la revue franco-allemande Apidologie dans l’espoir qu’elle verra le jour l’an prochain et avec mes remerciements au Prof. F. Ruttner pour son accueil.”

As editors, Jean Louveaux and Friedrich Ruttner experienced and often discussed those upcoming financial problems for the two journals. So, why not merge the two journals and attract subscriptions from both countries for a single journal? At the same time, they saw this as a step to further bring together French and German beekeepers and scientists. So, when Friedrich Ruttner organized a meeting on October 22, 1968, in Oberursel with Jean Louveaux and Oskar Wahl, the then director of the Bieneninstitut in Marburg and co-editor of the *Zeitschrift für Bienenforschung*, the decision to merge *Annales de l‘Abeille* with *Zeitschrift für Bienenforschung* was made. *Apidologie* was born! The name *Apidologie* was chosen, as it is the same word in both French and German (see the record in the guest book of Oberursel (Figure 2).

The next step was to convince and win INRA and the DIB as supporting institutions and owners for the new journal. These two institutions are, in fact, of quite different nature, as INRA is the French governmental institution promoting research in all areas of agriculture, while the DIB is the non-governmental organization of German beekeepers, and thus primarily devoted to promoting issues of interest to professional and hobby beekeepers. Nonetheless, in 1969, INRA and DIB signed a memorandum in Munich confirming their commitment to *Apidologie*, and then, already in the following year, the first issue of *Apidologie* was published, with Jean Louveaux as the French editor and Oskar Wahl as the German editor. Oskar Wahl was active as editor from 1970 until 1979, assisted by Friedrich Ruttner, who then replaced him in 1979 and assumed this position as German editor.

Academic editors need support in the daily journal business. So, since its beginnings, a crucial role in the *Apidologie* history was always that played by a Managing Editor. For decades, from 1971 until 2009, this position was held by Christiane Courant, who, back then had just joined INRA. The Managing Editor’s responsibilities were receiving and checking new manuscripts, passing them on to one of the editors, keeping contact with reviewers and authors, forwarding manuscripts to the publisher for production, and compiling the yearly journal issues. Those continue to be the tasks ever since, only the mode of communication has changed over the years. At the beginning, everything was done through postal mail, then by e-mail, and now via manuscript submission platforms.

**Figure 3.**
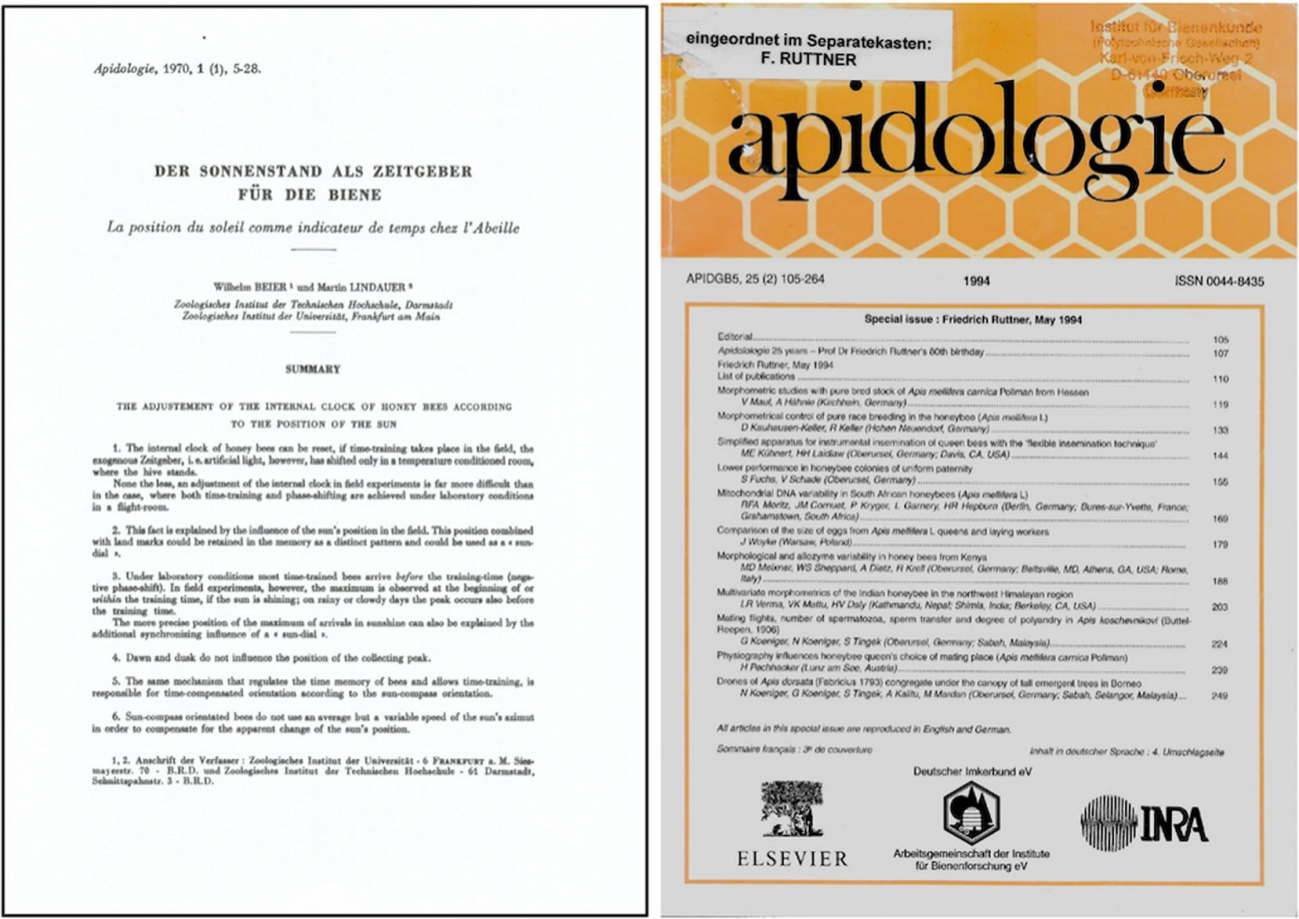
Images of keystone issues in the history of *Apidologie*. The image on the left is the title page of the first article published in the first issue of *Apidologie*. The image on the left shows the cover page of the special issue dedicated to Friedrich Ruttner on occasion of his 80th birthday. Coincidently, it is also the special issue of the 25th anniversary of *Apidologie*.

The first publisher, until 1986, was an “in house” one, *Le Service des Publications de l’INRA*, and already the first issue was remarkable, composed of six articles. Two were in German, one from Martin Lindauer on the adjustment of the honey bee’s inner clock in relation to the sun’s position (Figure 3), and the second one, coming from Gudrun Koeniger, was on the importance of the tracheal envelope for spermatheca function. The next two articles were in French, one by Janine Pain and Bernard Roger on 10-HDA levels in the head of honey bees, and the second by André Pouvreau on the hibernation of bumble bee queens. The last two articles were by Anna Maurizio and Jean Louveaux, and these were actually the French and German versions of the activity report of the International Commission for Bee Botany for the years1966–1969. Evidently, such double publication would nowadays warrant rejection because of plagiarism. But things were different back then, before the calculation of journal impact factors and h-indices.

As conceived by the two founders already from the beginning, *Apidologie* was to be an international journal, where authors had the option to submit manuscripts in French, German, or English. Let*’*s say options, as there were in fact certain restrictions that, in retrospect, seem anachronistic. For instance, Friedrich Ruttner insisted that German researchers, especially those from his home institution, had to publish their articles in German, based on the argument that *Apidologie* was supported by the DIB. This restriction was only removed when Stefan Fuchs and Gudrun Koeniger took over from Ruttner as German editors. It is not known whether such restrictions were also imposed by the French editor, but still for years, articles were published in either of these three languages, until in 2001, English became the only language for manuscript submissions. The solution to this trilingual configuration, was, for a long time, the addition of extended General Summaries at the end of each published article in the two additional languages. The idea was that this would also help to make the articles understood by less specialized readers. As these summaries were in languages other than those necessarily dominated by the submitting authors, it was frequently a job for the editors to translate these, and this was the case until 2006, when the production of trilingual General Summaries ended.

## The consolidation period

**Figure 4.**
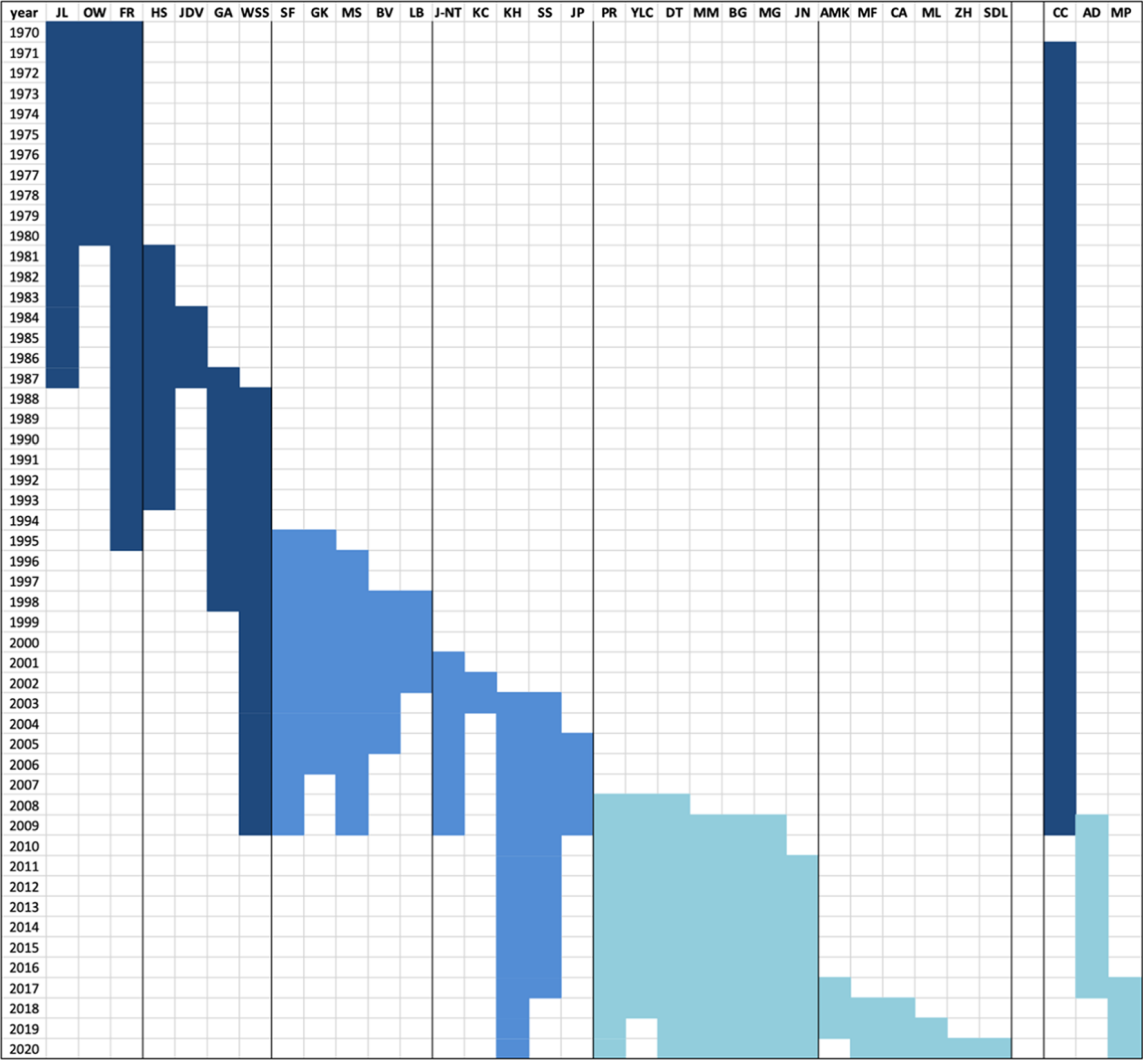
Editors of *Apidologie* from 1970 until 2020. The columns refer to the 28 Editorial Board members and the 3 Managing Editors, all identified by their initials. From left to right: JL, Jean Louveaux; OW, Oskar Wahl; FR, Friedrich Ruttner; HS, Hachiro Shimanuki; JV, John Vandenberg; GA, Gérard Arnold; WSS, Walter Steve Sheppard; SF, Stefan Fuchs; GK, Gudrun Koeniger; MS, Marla Spivak; BV, Bernard Vaissière; LB, Luc Belzunces; J-NT, Jean-Noël Taséi; KC, Karl Crailsheim; KH, Klaus Hartfelder; SS, Stan Schneider; JP, Jacqueline Pierre; PR, Peter Rosenkranz; YLC, Yves Le Conte; DT, David Tarpy; MM, Marina Meixner; BG, Bernd Grünewald; MG, Monique Gauthier; JN, James Nieh; AMK, Alexandra-Maria Klein; MF, Michelle Flenniken; CA, Cedric Alaux; ML, Mathieu Lihoreaux; ZH, Zachary Huang; SDL, Sara Diana Leonhardt; CC, Christiane Courant; AD, Anne Dufay; MP, Marianne Peiffer. Color codes depict the three major periods of the journal described in the text.

With the progressive internationalization of bee research in the 1970s and the increasing submission of manuscripts from countries other than France and Germany, especially from the USA, it became evident that an English-speaking editor was needed. It was then in 1981 that Hachiro Shimanuki, Head of the USDA Bee Research Laboratory in Beltsville, readily accepted to join the Editorial Team (Figure 4). He was assisted in this task by his colleague John D. Vandenberg.

With the entry of Hachiro Shimanuki and John D. Vandenberg, there were now *Apidologie* editors from three countries and three languages, and this continues to be so until today. All future editors were from one of the three countries due to the initial contract between INRA, DIB, and later the connection with the American Association of Professional Apiculturists (AAPA), even though the latter is not an owner of the journal. With this configuration of a now editorial trio, the first period and decade in the life of *Apidologie* effectively ended, and what comes thereafter is a continuing story of success of the journal.

In 1980, Oskar Wahl retired and Friedrich Ruttner took over as the sole German editor. At the Oberursel institute, he was assisted in the editorial duties by his former PhD student Dorothea Kauhausen. In 1985, he retired as the institute’s director but continued as editor of *Apidologie*, and he also had ongoing research projects until the end of the 1980s. The editorial trio Louveaux, Ruttner, and Shimanuki/Vandenberg continued to run the journal until 1987. Jean Louveaux retired in 1984 from his position at INRA but still continued as editor until 1987, when he was replaced by Gérard Arnold, also from INRA. In the same year, John Vandenberg stepped down and was replaced by Walter Steve Sheppard, who had just joined the USDA laboratory in Beltsville. These were the next-generation editors, who continued to run the journal together with Ruttner until 1995, when Ruttner stepped down (Figure 4).

Another major change that occurred during this period concerned the publishing and marketing of *Apidologie*, as in 1986, a bid was placed that was won by Elsevier France, and from 1987 until 1999, *Apidologie* was published by Elsevier, France. There are two curious side stories to this. The first one is that, different from what is practice nowadays, Elsevier France gave a champagne reception in the splendid *Grande Galerie de l’Évolution* of the *Muséum National d’Histoire Naturelle* in Paris to honor this contract. The second story is that the Elsevier director who then managed *Apidologie* sales changed the journal’s name to Apidology, thinking that this could help to increase sales in the USA. However, this was done without prior consent from the three editors, who strongly protested, saying that “Apidologie does not need a ‘y’ to be known among the English and American bee researchers.” This second phase of *Apidologie*, from 1981 to 1987, can, thus, justly be called the period during which *Apidologie* became consolidated as a leading journal that reports high-quality bee research, next to the *Journal of Apicultural Research*.

## The increase in bee research topics and expansion of the Editorial Board

The next decade, from 1995 until 2005, was marked by the entry of almost one new member per year into the Editorial Board (Figure 4). This was due to the increasing number of submissions on topics in bee research that had not been mainstream before the 1990s. These were reports on the biology of solitary bees, primitively eusocial bees, and also the highly eusocial stingless bees, in addition to manuscripts on the growing fields of bee molecular biology and bee/plant relationships.

As already briefly mentioned, in 1995, Friedrich Ruttner retired from the Editorial Board. His position was filled by Stefan Fuchs and Gudrun Koeniger, both from the Oberursel institute, and they promoted important changes in the journal’s business affairs. Especially so, they established a closer relationship with the German beekeepers association, DIB, and its scientific branch, AGIB. Gudrun Koeniger stayed on the Editorial Board until 2006. In 1996, Steve Sheppard gained company from Marla Spivak as new American editor, and between 1998 and 2001, three French editors, Bernard Vaissière, Luc Belzunces, and Jean-Noël Taséi from INRA, also joined the team. In 2003, the German editor Karl Crailsheim was replaced by Klaus Hartfelder, Stan Schneider entered the Board as a new American editor, and on the French side, Jacqueline Pierre joined in 2005 for a period of 5 years.

During this period, another novelty was the creation of an International Scientific Board, to which renowned bee researchers were invited on a rotating basis. Their help is frequently requested by the editors in cases of critical issues, for instance in cases of strongly contrasting reviewer recommendations on manuscript acceptance or rejection. Invitations to the International Board are decided during the annual meetings of the Editorial Board.

According to the contract between INRA and DIB, such annual meetings have to be held to discuss editorial policies and also business matters between the journal owners INRA and DIB, and the publisher. These yearly meetings usually alternate between Paris at INRA and the Bee Research Institute in Oberursel near Frankfurt/Main. The only exceptions were in 2016, when the meeting was held in Sophia-Antipolis, near the former Avignon editorial office, and now in 2020, when the coronavirus pandemy COVID-19 made it necessary to have a virtual meeting.

These meetings are well within the working spirit of the journal’s founders Louveaux and Ruttner who, in the *Apidologie* charter, had fixed that there would be no Editor-in-Chief, as is the case for most scientific journals, but an Editorial Board with equal collegial functions. Perhaps the best formulation of the philosophy behind *Apidologie* was given by Christiane Courant in 1998, on the occasion of a symposium in honor of Friedrich Ruttner: “Apidologie, c’est en fait une belle histoire d’amitié qui se moque des frontières. En ces temps de compétition exacerbée, dans un monde où l’être humain est bien souvent broyé, il est heureux de trouver un lieu où efficacité et réussite savent rimer avec humanité.“ “Die Apidologie beruht im Grunde auf freundschaftlichen Beziehungen, die sich über die Grenzen hinwegsetzen. In einer Zeit von verschärfter Konkurrenz, in einer Welt, in der der Mensch oft genug zermalmt wird, ist es schön, einen Ort zu finden, wo sich Leistung und Erfolg mit Menschlichkeit reimen.”

While democratic, on the one hand, this required an intense exchange of information among the editors, while on the other hand, the communication on submissions and the respective decisions on acceptance or rejection turned out to be an important platform to maintain the journal’s cohesiveness with regard to scope and its projection within the bee research community. This flux of manuscripts and information meant, of course, a lot of work for the Managing Editor, Christiane Courant, especially while still done via postal mail and later e-mail. A big help for her was the implementation of an electronic submission platform offered by the publisher EDP Sciences, but of course, this platform first had to be configured to match *Apidologie*’s communication flow chart, and then the editors had to be trained. EDP Sciences (Édition Diffusion Presse Sciences), a small, learned societies publisher with its headquarters in Les Ulis, France, had won the bid to publish *Apidologie* after the contract with Elsevier had ended. EDP Sciences did not throw a champagne reception, as this was then out of fashion but was an important companion for *Apidologie* until 2010, providing assistance to the Managing Editor and organizing the marketing and subscription sales.

As important as sales are, the main asset for a journal is its attractiveness to the field, both to authors and readers, and to further this, the editors promoted and organized, frequently with the help of invited guest editors, the publication of Special Issues presenting reviews and original research articles on timely topics in bee research. This policy was adopted already in 1988, resulting in the publication of by now 22 special issues on a yearly/biannual basis. A list of all these is compiled in Table I. The special issues and also the regular volumes from 1970 to 2010 are all freely accessible at https://www.apidologie.org/. Those after 2010 can be accessed at https://link.springer.com/journal/13592. These special issues include a particularly remarkable one dedicated to the 80th birthday of Friedrich Ruttner (Figure 3).

**Table I:** Special issues published in *Apidologie*. The special issues from 1989 to 2010 are all freely available at https://www.apidologie.org/component/issues/?task=special. Those from 2010 to 2019 are available at https://link.springer.com/journal/volumesAndIssues/13592

Year	Issue	Title	Articles
1989	20,5	Genetics	9
1990	21,5	Anna Mauricio - honeys	8
1991	22,6	Evolution and genetics	7
1993	24,3	Neurobiology of the honeybee	11
1994	25,2	F. Ruttner 80th birthday	11
1995	26,3	Non-Apis bees	7
1996	27,5	Asian honeybees	12
1997	28,5	Intra-specific variation in the honeybee	7
1998	29,1	Colony integration	11
1999	30,2	Dynamics and control of Varroa parasitism on Apis	12
2000	31,2	Taxonomy and evolutionary biology of the honeybees	13
2002	33,2	The Cape honeybee (*Apis mellifera capensis*) from laying workers to social parasites	10
2004	35,1	European unifloral honeys	8
2004	35,2	Information flow and group decision making in social bees	10
2005	36,2	The neglected gender – males in bees	12
2006	37,2	Stingless bees: biology and management	12
2008	39,1	Insights into bee evolution: a tribute to Charles D. Michener	14
2009	40,3	Bee conservation	15
2010	41,3	Honey bee health	14
2012	43,2	Current concepts in honeybee neurophysiology	9
2014	45,3	Mechanisms of social evolution	7
2016	47,3	A new perspective on honey bee health	11
2018	49,1	Molecular signatures of phenotypic and behavioral plasticity in bees	6
2020		*Apidologie* 50 years	-

Actually, all issues of *Apidologie* since 1970, as well those of its precursor, the Annales de l’Abeille (1958–1968) are freely available from INRA’s HAL server (https://hal.archives-ouvertes.fr/ARINRA-APID/; https://hal.archives-ouvertes.fr/ARINRA-ABEILLE/).

## 2009 until 2020, a new Editorial Board and a new publisher

As in 2008/2009, several of the long time Editorial Board members, Steve Sheppard, Stefan Fuchs, Marla Spivak, as well as Jean-Noël Taséi and Jacqueline Pierre, had announced their retirement from the Editorial Board, an intense search for new members became necessary. As shown in Figure 4, Peter Rosenkranz, Marina Meixner, and Bernd Grünewald, the current Director of the Oberursel Bee Institute, entered as new German editors, Yves Le Conte from INRA and Monique Gauthier as new French editors. David Tarpy, and slightly later in 2011, James Nieh became the new American editors. These now joined the only two remaining editors from the former period, Stan Schneider and Klaus Hartfelder.

**Figure 5.**
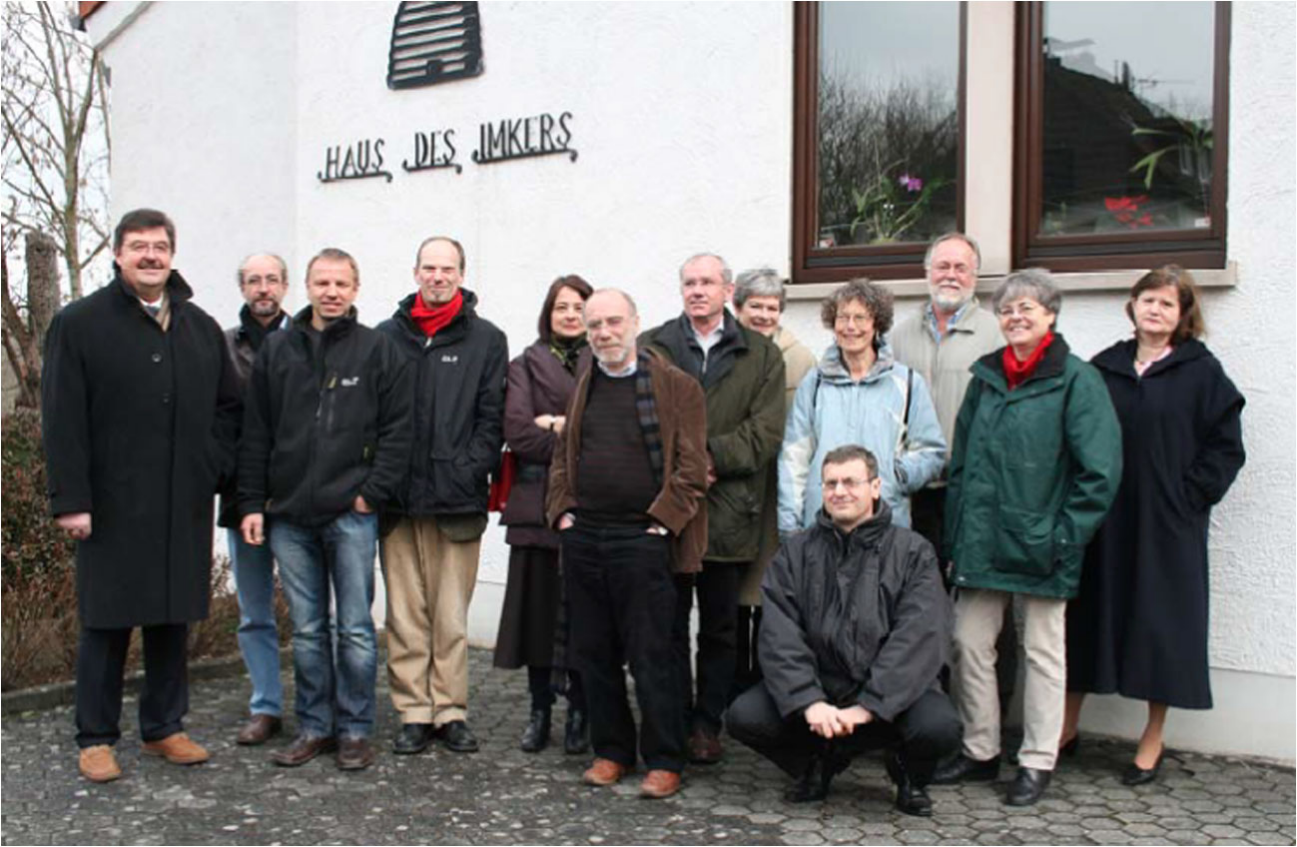
*Apidologie* Editorial Board meeting 2009. The picture was taken in front of the headquarters of the German Beekeepers Association (DIB) in Wachtberg-Villip, near Bonn, Germany. From left to right: Peter Maske (DIB), Klaus Hartfelder, Peter Rosenkranz, Bernd Grünewald, Agnes Henri (EDP Sciences), Stefan Fuchs, Yves Le Conte, Barbara Guérout (translator), Christiane Courant, Pierre Castelli (INRA, front row), Steve Sheppard, Anne Dufay, Barbara Löwer (DIB).

The most crucial challenge in 2009, however, was the replacement of Christiane Courant as Managing Editor (Figures 4 and 5). She was an INRA staff member since 1971, heading the *Apidologie* Editorial Office in Avignon, so a long negotiation with INRA was necessary to find and appoint a substitute for her among the INRA staff, and this was Anne Dufay. In hindsight, this was probably one of the really decisive steps that kept the *Apidologie* ship afloat.

This composition of the Editorial Board and Managing Editor continued until 2017, when increasing submission numbers and new topics, especially bee-plant relationships, landscape ecology, and more and more molecular approaches to all kinds of bee research required a search for additional editors. In 2017/2018, these were Alexandra-Maria Klein, as German editor, who unfortunately could fulfill this task only until 2019, Michelle Flenniken as American Editor, replacing Stan Schneider. In 2018, Cedric Alaux from INRA, and then in 2019, Mathieu Lihoreau entered the team as new French editors, and now, in 2020, Zachary Huang and Sara Diana Leonhardt filled gaps in the team as new US and German editors, respectively. Furthermore, Anne Dufay retired in 2017, after serving for 9 years as Managing Editor. Again, INRA agreed to find a replacement for her, so as of 2017, the Managing Editor position is now held by Marianne Peiffer at the INRA center in Champenoux near Nancy.

Another change that came in 2010 was the end of the publisher contract with EDP Sciences. EDP Sciences had been a supportive publisher, providing important editorial support, such as the journal’s first online submission platform, and conducting a marketing strategy that guaranteed the journal’s financial stability. This was in times when the publishing of scientific journals underwent major changes, with the increase in the number of online-only electronic journals (eliminating printing and shipping costs) and Open Access as a new business model, alternative to the traditional, subscription-based model.

Financial resources were declining for institutional libraries, so they could afford less and less the journal subscription fees, which, at the same time, increased with the growing monopolization among scientific publishers. The movement towards Open Access, with the immediate availability and general accessibility of scientific data also for non-subscribers, put the financial burden for publishing now on the shoulders of the scientists and the institutions that financed their research. *Apidologie* kept its print version together with the already available online version. It also adopted a hybrid business model, continuing the subscription-based journal sales and, at the same time, offering authors to have their articles immediately accessible to all public through payment of an Open Access fee. This business model was implemented by EDP Sciences, and as of 2011, continued as part of the contract with the new publisher, Springer Netherlands, which had won the bid in 2010 for publishing *Apidologie* until 2020. Independent, however, of which company is the publisher, it is no secret that what keeps a specialized journal healthy, both financially and scientifically, is a solid editorial policy that seeks quality over quantity.

Scientific quality first has been a commitment of *Apidologie*’s Editorial Board since its beginnings, and in this quest, the editors strongly depend on *ad hoc *reviewers for the analysis of submitted manuscripts. These are the unsung heroes behind the scenes in every serious journal, and even more so, they do this for free, as part of their dedication to science. Hence, here we now take this unique opportunity to thank all the reviewers for their invaluable input to *Apidologie* along these many years.

With this, we end our excursion into the history of *Apidologie* and, returning to its French-German origin, what could be more appropriate to say than these two sentences to summarize the 50 years of *Apidologie*: “Fünfzig Jahre sind kein Honigschlecken” and “Cinquante ans ce n’est pas une mince affaire” (50 years is not a honey lick/50 years is not an easy task).

